# Embodied leisure experiences of nature-based activities for people living with dementia

**DOI:** 10.1177/14713012241262384

**Published:** 2024-07-26

**Authors:** Steven Owen, Stephen Page, Katie Ledingham, Stephan Price, Joanne Connell, Catherine Quinn, Linda Clare

**Affiliations:** 102002 University of Exeter Business School, UK; 3769 University of Hertfordshire, UK; 102002 University of Exeter Business School, UK; Centre for Applied Dementia Studies, 1905 Bradford University, UK; Wolfson Centre for Applied Health Research, Bradford, UK; 3286 University of Exeter Medical School, UK; NIHR Applied Research Collaboration South-West Peninsula, UK

**Keywords:** the outdoors, embodiment, everyday life, Alzheimer's disease, qualitative research

## Abstract

**Purpose:**

This article adopts an embodiment lens to explore the individual leisure experiences of people living with dementia when engaging in nature-based pursuits. It focuses on how people living with dementia frame their everyday experiences of nature and how these are shaped by any cognitive challenges and/or other comorbidities affecting physical health.

**Design/methodology/approach:**

Taking a phenomenological research approach, we interviewed 15 people living with dementia and 15 family carers of people with dementia to explore how people with dementia engage with nature as a subjective leisure experience. We analysed their accounts using reflexive thematic analysis.

**Findings:**

The findings reveal how people living with dementia frame their experiences of nature-based pursuits through three interlinked themes of ‘bodily feelings and emotions’, ‘sense of self and identity’ and ‘connectivity to others’.

**Originality/value:**

The paper contributes to knowledge by examining through the lens of embodiment a neglected and overlooked dimension of everyday leisure: how nature is encountered, negotiated and enjoyed. The paper illustrates how nature and the outdoors may help people living with dementia to continue to enjoy prior leisure pursuits and thus achieve a degree of continuity in their everyday lives.

## Introduction

This paper uses the lens of embodiment to explore how enjoyment of nature and greenspace contributes to improving, supporting, and maintaining health and well-being for people living with dementia (Martin et al., 2020; Twohig-Bennett & Jones, 2018) in their leisure time. Leisure as a concept has a long history of analysis within social science (Page & Connell, 2010), typically defined in simple terms as the time left after an individual has fulfilled all of their daily obligations and duties. The scope and breadth of leisure was defined by Stockdale (1985) who identified three main ways in which the concept is used:

 • *as a period of time*, activity or state of mind in which choice is the dominant feature;
 • *an objective view* in which leisure is perceived as the opposite of work and is defined as non-work or residual time;
 • *a subjective view* which emphasises leisure as a qualitative concept in which leisure activities take on a meaning only within the context of individual perceptions and belief systems and can therefore occur at any time in any setting (Hall & Page, 2014, p. 7).

For people living with dementia, conceptualising leisure is slightly more complex because of the need for care and the time that providing care may take up as a daily necessity whether by an unpaid family member or friend acting as an informal carer or by a paid care worker. This also has the potential to alter the social dynamic of the relationship that is perceived to exist between the person living with dementia and the carer in leisure settings.

In this paper, our emphasis is upon the *subjective view* of leisure in relation to people living with dementia, recognising the growing interest in leisure and everyday life (Gray et al., 2024) as well as the diverse ways in which leisure time experiences are constructed, consumed and mediated through different social and psychological factors. As Gray et al. (2024) suggest, people living with dementia each have a unique experience of living with the condition, and by implication, their everyday experiences of life and dementia are equally individualised and nuanced. For this reason, the paper also provides a series of insights on social citizenship and leisure in everyday life with dementia, because engaging with nature and the outdoors is inherently about how individuals sustain a sense of place in their world through what they do (Bartlett & O’Connor, 2010) and the meaning derived therein. As Gray et al. (2024) suggest, these leisure realms that are both space- and place-related offer freedom for people living with dementia that enhances their well-being. This is important for the psychological health of people living with dementia in terms of self and identity (Caddell & Clare, 2013; Clare et al., 2020) as it reflects how individuals create subjective evaluations of self and identity through the tripartite model of Sabat & Harré (1992). This model is based upon three components: personal identity, beliefs and attributes, and the personae presented in social interactions (Clare et al., 2020, p. 127). Engaging with nature and the outdoors may enhance individual feelings of self and help people living with dementia to maintain a degree of continuity in their leisure lives, thereby achieving a sense of phenomenological continuity. In addition, Russell et al. (2023) argued that social identity was important for people living with dementia as leisure was one way in which they could be viewed as people rather than being defined by their medical condition. In this respect, leisure activities and participation have a pivotal role to play in social identity through the way in which social interactions and their engagement with leisure-oriented places and spaces can be understood phenomenologically through embodiment and ‘being in the world’ (Russell et al., 2023, p. 136).

Positive enhancement of well-being through engagement with nature can also help reduce depression and anxiety, and improve cognitive function, mood and quality of life (QoL; see e.g., Edwards et al., 2013; Finnanger-Garshol et al., 2022; Hewitt et al., 2013; Zhao et al., 2022), while Odzakovic et al. (2020) and Ward et al. (2022) have emphasised how natural settings support a sense of self and well-being through their restorative effects and opportunity for enhanced sociability, creating a positive ethic about living well with dementia (Bartlett et al., 2017). Further, this paper addresses the dearth of studies on the leisure-dementia nexus by examining the leisure lives of people living in the community (an exception being Gray et al., 2024) rather than in residential care settings, the context in which the role of nature has been more frequently studied (Cook, 2020; de Bruin et al., 2021; Gibson et al., 2017; Noone & Jenkins, 2018; Whear et al., 2014). Leisure participation may empower people with dementia to resist and overcome the stigma of a dementia diagnosis (Genoe, 2010) and it offers a personal space where friendship can flourish, reducing social isolation (Fortune et al., 2021). Research on individual subjective leisure experiences of dementia (Genoe & Dupuis, 2014) that incorporate nature is limited to pilot studies (e.g., Innes et al., 2016) but given that most people with a dementia diagnosis live independently at home (Mmako et al., 2020), there is a need to better understand what facilitates independent outdoor nature activities. This is because there is a very clear knowledge gap in how engaging with the outdoors and nature may boost sense of identity as exhibited through place attachment, which is the emotional bond an individual has to a particular place(s). This connects with the inclusivity paradigm that suggests access to leisure resources, such as nature, must recognise individual capability, barriers to participation and diversity in needs and preferences. However, as Collins et al. (2023) emphasised, the research focus on nature-based activities for people living with dementia tends to be formalised through green day care, equine-assisted interventions, and community nature-based activities. In this way, people living with dementia are often overlooked in terms of their leisure habits, aspirations, behaviours and activities and the resulting cognitive challenges faced in outdoor environments, particularly spatial awareness and wayfinding (see Duggan et al., 2008). Mapes et al. (2016, p. 50) argued that we need to understand how people with dementia “conceptualise, experience and interact” with natural environments (see Table 1) to fill the knowledge gap on how people living with dementia engage with nature. Yet, understanding the meaning, value and overall experience of nature to date has largely focused on structured leisure activities rather than independent experiences of leisure in daily life.

**Table 1.** Summary of findings from Mapes et al. (2016).

Where like to go in nature	• Places near water	• Allotments		
	• City parks	• Inland waterways/Lakes		
	• Beach/Coast	• Woods		
	• Country parks	• The wilds		
What like to do in nature	• Walking informal	• Gardening		
	• Wildlife watching/Interacting with nature	• Boating		
	• Dog walking	• Swimming		
	• Organised walking group	• Bushcraft		
	• Cycling	• Running		
Influencing preferences	• Where live (urban/rural)			
	• Concept of nature (present day experience, memory, emotional and idealised view of nature)			
Influencing engagement with nature	Personal attributes/feelings	Facilities	The support of others	Activities
	• Difficulties with mobility and tiredness, a fear of getting lost, concerns about the reactions of others, a feeling of being a burden to others or of not liking to ask for help, Fear of crime. • A lack, or loss, of confidence (carer observation). • Sensitivity to cold (carer observation) • Moods or behaviour (carer observation) • Cultural issues (carer observation)	• Transport • Signage, navigation, orientation • Information • Refreshments • Physical access	• Individuals • Organisations	• Availability of activities

The report by Mapes et al. (2016), commissioned by Natural England, presents evidence about engagement with the outdoors and with nature in the UK gathered from 54 people living with dementia via focus groups and 182 informal carers via online survey or telephone interviews.

This paper aims to explore embodied experience of nature-based and outdoor activities among people with dementia and their unpaid, informal carers, in this case spouses/partners or adult children, collectively described as ‘supporters’. In doing so, we draw on the concept of embodiment, as through embodiment ‘the individual comes to know about the world in new, more complex ways…in everyday activities the individual works and reworks’ (Crouch, 2000, p. 65). Furthermore, people encounter place, space and leisure resources, including nature, through embodiment. It is a powerful conceptual frame to examine dementia as the “body and embodiment are central to the experience of living with dementia…[by]… heightening of embodied selfhood in dementia; recognising … the enormous potential that embodiment offers us to celebrate life with dementia” (Downs, 2013, p. 372). Embodiment as a concept enables researchers to understand and create an account of the sensual elements of place, space and nature in multi-dimensional and multi-sensual ways, typically focused on the moment or element of an experience.

## Methodology

### Design

This study presents qualitative interview data from ‘Extending active life for older people with cognitive impairment and their families through innovation in the visitor economy of the natural environment’ (ENLIVEN), which was funded by the UK Research and Innovation (UKRI) Healthy Ageing Challenge and Economic and Social Research Council (ESRC). Ethical approval for the study was provided by the University of Exeter, Reference 51316. ENLIVEN is a cross-institutional, multi-partner programme working to co-produce dementia friendly innovations in the visitor economy. The project has been focusing on increasing access to natural environments for older people experiencing cognitive impairment.

### Methodological approach

We adopted a phenomenological approach that aimed to understand the lived experience of individuals and uncover how this experience is given meaning (Chamberlain, 2009). Exploring experiences in a meaningful way requires a participant-focused methodology that captures the voices and feelings of people living with dementia. Theoretical studies of leisure geographies (e.g., Crouch, 2000) point to the importance of subjective research methodologies to understand how people create an account of place in their everyday lives, including the role of nature. Embodiment offers one such approach (see Spatz, 2017), focusing on “(1) Social theories of the body; (2) ‘Histories’ of the body; (3) Analyses of bodily techniques; (4) Studies of embodied experience” (Brown et al., 2011, p. 493). Embodiment offers a reframing of dementia away from cognitive decline and loss to explore the unique way individuals experience and construct meaning through their body (Kontos & Martin, 2013; Millett, 2011). Embodiment explores “the ways in which individuals grasp the world around them and make sense of it in ways that engage both mind and body” (Crouch, 2000, p. 63) and has been widely used as a research approach in cultural and leisure geographies, and more widely in social science. Embodiment helps understand people’s connection to place and space as well as helping reposition the leisure and ageing research agenda towards care for oneself and care for the other (Rojek, 2005), sharing a theoretical congruence with dementia research. Embodiment rejects the separation of the mind and body, recognising their interconnectedness in shaping how humans experience the world around them, which “repositions the body, as a source of knowledge and understanding, as opposed to simply a vessel for the mind” (Fleetwood-Smith et al., 2022, p. 265). Embodiment helps understand resistance to dementia through leisure (and access to nature), enabling people to exhibit personhood and notions of self through their bodily responses, feelings, and movement (Downs, 2013; Isene et al., 2022; Martin et al., 2013; Millett, 2011).

Previous studies of dementia and leisure have adopted an embodiment lens to examine structured and organised experiences of people living with dementia (Dowlen et al., 2022; Kontos et al., 2021; Robertson & McCall, 2020; Scott, 2019; Wright, 2018). These studies evaluated how familiar activities may instigate emotional and bodily responses to facilitate self-expression and enhance social connectivity with others (Russell et al., 2023), typically with an instrumentalist approach of monitoring pre and post health indicators following leisure activity. A few studies have extended this focus to nature-based experiences. Noone and Jenkins (2018) found nature provided embodied selfhood (see Kontos, 2012), opportunities for self-expression, autonomy, and connecting to others. Smith-Carrier et al. (2021) noted the pleasurable, sensuous experiences of a therapeutic gardening programme, generating feelings of reminiscence, accomplishment, and enhanced social connections. Cook (2020) observed that multi-sensory experiences in nature instigated feelings of autonomy and identity whilst also enhancing social interactions.

### Participant recruitment

Participants were recruited through partner organisations, the online portal Join Dementia Research, and the team’s existing contacts. Potential participants were older community-dwelling individuals who identified themselves as living with dementia or cognitive impairment, as well as family carers and supporters of people with dementia (either living or deceased). Participants needed to have the capacity to provide informed consent. Thirty participants were recruited, equally distributed amongst those with dementia and carers or supporters. The participants included one dyad consisting of a person with dementia and a supporter; the rest were independent of each other. Of the 15 people living with dementia interviewed, the majority were male (10) and White British (12) and all except one was over 55 years old. The mean age was 70 (range 52 – 91; standard deviation 11). Of the 15 family members/supporters interviewed, the majority were female (10) and White British (9) and all except one were over 55 years old. The mean age was 67 (range 55 – 90; standard deviation 10).

### Interviews

Interviews took place between November 2021 and March 2022, and were conducted via telephone or video call largely because the study was undertaken during the COVID-19 pandemic. Each interview was conducted after an information sheet had been shared with the participants and informed consent was given, and interviews lasted up to an hour. To create an interview schedule sensitive to the needs and ability range of people living with dementia, topics were considered by an advisory group consisting of a panel of people with dementia. Most questions were semi-structured to allow for exploration of participants’ views, and the focus was on nature, the outdoors and leisure experiences. Participants’ sensory experiences of nature, their emotions and feelings, and the ways in which they liked to experience nature were explored. Photographs of older people engaging in a range of nature-based pursuits were used to aid discussion of participants’ interests.

### Data analysis

To draw out the essence of participants’ embodied experiences, reflexive thematic analysis was used. As Braun and Clarke (2021) advocate, the six phases of reflexive thematic analysis were followed when analysing the data: familiarising with the data; generating codes; constructing themes; reviewing potential themes; defining and naming themes; reporting the findings. Researchers read the transcripts multiple times to familiarise themselves with the data. During this process the importance of the body within the transcripts was identified. Using QSR NVivo1.6.1, the primary author coded the transcripts in relation to the body generating initial themes. A selection of transcripts was also independently coded by two researchers. Through discussion and negotiation, authors refined the themes until they were satisfied that these accurately captured the experiences of participants engagement with nature-based pursuits. These themes are explored in the next section.

## Findings

Participant characteristics and preferences in visiting nature sites are shown in Tables 2 and 3; participants’ names have been pseudonymised. Self-reported health issues and physical limitations identified by participants, and how these affect their engagement in nature-based activities, are also shown in Tables 2 and 3. Table 2 outlines the nature-based interactions occurring in the everyday lives of participants which, in broad terms, do not vary significantly from leisure activities among the wider population (ONS, 2017). ONS identifies that people over age 65 (and the long-term sick) have potentially in excess of 7 hours a day of leisure time available but this latent time must be translated into meaningful leisure time by engaging in activities.^1^ Such latency is a fluid concept for people living with dementia due to the variability in how cognitive abilities diminish and time-space compression emerges in the lived experience of leisure. For some people, this can create a feeling of disappointment about restrictions felt or experienced in getting out into greenspace. Conversely, others highlight a desire to notice, appreciate, and take pleasure from nature to resist the condition (Genoe, 2010).

**Table 2.** Profile of interview participants - People living with dementia.

Name (Pseudonym)	Ethnicity/Nationality	Location	Supported by	Self-identified difficulties	How affected	Preferences
Tom (Male 70)	White/British	Midlands	Lives with wife	Remembering names. Multi-tasking. Hearing impaired. Feet desensitised and painful. Balance (uses walking stick). Diabetes. Heart by-pass	Avoids steep hills/mountains. Tries to avoid going out when windy and cold. Tires quickly. Wind disorientating	Walking on the beach. Bird watching. Walking dog. Local circular walk
Paul (Male 67)	White/British	Midlands	Lives with wife	Recent and some long-term memory. Balance. Depth perception	Unable to walk by self to go metal detecting. Cannot do heavy digging so no longer has an allotment. Gets cold quickly so cannot stay out long in cold weather for fishing or walking.	Metal detecting. Fishing. Walk around local park. Bee keeping with son. Managing small back garden. Walking around garden estates. Foraging. Travelling abroad.
				Spatial awareness.		
				Word searching (depending on day). Stroke - on blood thinners. Arthritis		
				Hip/shoulder replacement		
Henry (Male 52)	British	South-West of England	Lives with girlfriend	Recent memory. Forgets names. Spatial awareness	Recent memory – only go familiar places or with someone else.	Walking in woods. Going for long walks. Open water swimming. Running. Cycling.
David (Male, age not reported)	White/British	South-East of England	None	Damaged tendon in foot. Balance/spatial awareness. Perception and sensory. Heart attack. Word finding.	Limited in distance can walk and struggles with uneven surfaces.	Visiting castle grounds. Being near water. Visiting local park with lake. Sport England walks. Walking. Travelling in UK.
					Advised to stop cycling for safety. Unable to go camping and fishing. cannot pull over a fence or wander across a ploughed field -avoids out of the way places. Always has phone.	
Kelvin (Male 70)	White/British	Northern England	None (family/friends support)	Recent memory. Spatial awareness. Balance. Registered deaf	No longer able to navigate yacht. Struggles setting up bait for fishing prefers to go with someone else. Avoids walking when windy.	Sailing. Fishing. Camping. Walking. National Trust site. Coast. Foraging. Travelling UK/abroad.
				Some hallucinations.		
Sarah (Female 70)	White/British	Midlands	Husband/walks with daughter	Recent memory. Mobility issues (a condition that is like MS)	Less confident prefers to walk with someone else.	Walking around lanes with daughter. Travelling UK/abroad.
Mathew (Male 90)	White/British	Northern England	None (son lives locally)	Recent memory	Does not go out in rain or wind. Cannot do major digging in garden (has a gardener to help).	Circular walks in countryside. Walking group. Gardening. Travelling UK with family. Looking at views.
					No longer drives so cannot get to some walks used to do.	
John (Male 66)	White/British	Northern England	Lives with wife	Recent memory. Stroke – left side weakness	No longer drives so cannot go fishing.	Walking dog. Walking group. Gardening with men’s group
					Worried might fall over – Goes places with family.	
Peter (Male 62)	White/British	Northern England	None	Balance issues – uses walking stick. Misplaced memory. Restricted movement in hands. Effected by light shining of water	Avoids going up hills. Avoids going to remote places as no rest places. Sticks to familiar places. No longer able to go fishing by self – struggles tying hooks and affected by ripples on water	Walking. Walking groups. Interacting with animals. Travelling local region.
Caroline (Female 76)	White/British	South-West of England	Lives with husband	Recent memory. Struggle with depth perception. Stiffening joints. Sensory difficulties.	Worries about getting lost carries a map and phone.	Walking in woods. Cycling. Foraging. Visiting gardens. Outside group (various activities). Travelling UK/abroad.
					No longer can lead groups pulling invasive weeds from river.	
					Prefers to use walking poles to help with depth perception.	
Lucy (Female 91)	British	South-East of England	None	Memory difficulties. Mobility issues – walks with a stick. Injured knees. Injured neck. Lack of movement in arms. Hearing impaired	Only goes to familiar places or with someone else. No longer able to cycle. Avoids walking groups so not slow others down. Cannot garden as struggles to bend (has a gardener)	Trips with university of 3rd age. Looking at views. Walking local parks. Watching wildlife.
Samantha (Female 66)	White/British	Midlands	Lives with partner	Recent memory. Over sensitive hearing. Mood swings.	Only runs familiar routes when by self – fear of getting lost.	Running. Walking around local fields. Walking. Visiting Brecon. Travelling UK/abroad
Robert (Male >60)	Middle Eastern	North-East of England	Lives with wife	Memory difficulties. Speech difficulties. Back pain/leg pain. Mobility issues.	Due to back can only walk 15–20 minutes. Sticks to familiar route. Family monitors through app.	Walking dog. Travelling UK/abroad. Foraging.
Mia (Female, age not reported)	British	South-West of England	None (daughter lives close)	Memory difficulties. Joint pain. Struggles bending. Sore painful wrist	Struggles going over stiles. Only goes to familiar places when by self. Struggles gardening	Walking dog forest. Walking dog local fields. Seeing hearing wildlife.
James (Male 70)	White/British	Midlands	Lives with wife	Memory difficulties. Hearing impaired	Only plays golf with people knows well. Gets tired quicker now	Playing golf. Walk around woods. Fishing. Walking around cemetery. Gardening.

**Table 3.** Profile of interview participants – Supporters of people living with dementia.

Name	Nationality/Ethnicity	Location	Relation to person living with dementia
Olivia (Female 75)	British	South-West of England	Spouse (husband living with dementia)
			Husband – 90
			Live together
Noah (Male 72)		South-West of England	Spouse (wife living with dementia.)
			Wife – 72
			Live together
Emma (Female 64)	British	South-West of England	Daughter (mother living with dementia)
			Mother – 87
			Lives in privately owned sheltered accommodation (carer comes morning and evening)
			Daughter lives 20 minutes away.
Charlotte (Female 57)	White/British	South-West of England	Daughter (father living with dementia)
			Father – 86
			Father lives by self (carers morning and evening)
William (Male 76)		South-West of England	Spouse (wife – deceased was living with dementia)
			In a care home 3 years before deceased
Amelia (Female 70)	White/British	Midlands	Spouse (husband living with dementia)
Lucas (Male 58)	White/British	Northern England	Son (mother – deceased was living with dementia)
Ava (Female, age not reported)		East of England	Daughter (father – deceased was living with dementia)
			Lived alone/then care home
Wyatt (Male 69)	White/British	London	Spouse (Wife living with dementia)
			Lived with wife. She is now in a care home.
Sophia (Female 60)	White/British	Midlands	Daughter (both parents – deceased were living with dementia)
Luke (Male 90)		Northern England	Spouse (wife – deceased was living with dementia)
Isabella (Female 55)	Black/African	London	Daughter (father – deceased was living with dementia)
			Lived 5 min away from father
Scarlett (Female 70)	White/British	Northern England	Wife (husband living with dementia)
Nora (Female 56)	White/British	South-West of England	Daughter (mother living with dementia)
			Mother 82
			Lives 15 min away from mother
Hazel (Female 62)	White/South American	London	Partner (partner living with dementia)
			Live together
			Partner 78

Three themes were identified in participants’ accounts: ‘*bodily feelings and emotions*’, ‘*sense of self and identity*’ and ‘*connectivity to others*’. These are discussed in turn below.

### Bodily feelings and emotions

Participants’ narratives exhibit positive feelings in engaging in nature-based activities, and these feelings emerge through and by the interconnected cognitive, sensate, and corporeal body (i.e., how meaning about the body and identity is mediated by culture and society). Feelings expressed in relation to nature include those of ‘freedom’, ‘calmness’, ‘enjoyment’, ‘excitement’, ‘peacefulness’, and an overall feeling of ‘being recharged’ from an initial analysis of the narratives. These narratives ideally need to be read and understood in the context of each individual’s characteristics and the multiple ways in which feelings are invoked through and by the body. A cornucopia of sensuous experiences result, as these quotations illustrate:

“It’s just so relaxing. It’s the open fresh air I love – to me it’s hearing birds and stuff… and then you’ve got the smells, you know if somebody’s got cattle or there’s horses or something, it’s the different smells… when people say to me it’s raining what are you doing going out? I’ve just got to feel the rain on my face and the wind. It makes me feel alive”. (Peter – living with dementia)

“It can be quite mindful when you go for a walk and enjoy just listening to, or looking at, the leaves on the trees and just sort of take a moment.” (Henry – living with dementia)

For Peter and Henry, tactile, auditory, and visual experience of nature resonate throughout the body, instilling feelings of enjoyment and rejuvenation, and mindfulness expressed as ‘the moment’ (see Djernis et al., 2019). Sensory stimulation through nature was valued by participants, who felt it was good for the body and the mind, with perceptions of individual agency in enhancing one’s own well-being, illustrating many of the arguments from positive psychology:

“You think you’re doing yourself a bit of good by being out and walking and just listening to everything that’s going on” (Sarah – living with dementia)

“When I realised what was happening with my wife, I was very focused on keeping her safe, keeping her stimulated, keeping her fit and all of those things that fit into being outdoors…I think that degree of stimulation and happiness maybe just kept the dementia at bay a little bit longer than would perhaps otherwise have been the case.” (Wyatt - Supporter)

These quotations illustrate a deeper meaning beyond sensory stimulation, also connecting with the happiness-dementia nexus (Person & Hanssen, 2015) through shared experiences. An underlying connection made by several participants was slowing the progression of dementia through nature, with the sensate, corporeal, and cognitive body experiencing and being shaped by nature. Some participants deliberately slowed or stopped their bodily movement to enhance their sensuous experience and connection to nature, again reiterating the mindfulness concept and enjoying the moment in full:

“When I walk, sometimes I will just stop where I am and just drink in, if you like, the atmosphere and the beauty that’s there, and the silence it’s a good feeling. It’s a warm feeling, you know, like a positive feeling.” (Samantha – living with dementia)

Samantha demonstrates the positive emotions experienced through immersing her sensate body in nature and connecting to her surroundings. Similarly some participants appreciate being alone in nature or walking in silence. For example, walking on the beach with his wife Tom describes his bodily movement as one that allows for a deep connection with his surroundings:

“I prefer to walk in silence. I’m a person who just wants to look and take it all in and think and not talk. I want to absorb it. So, when we went to the beach the other day, we didn’t talk most of the time, we just walked and meandered on our own different ways and picked up shells and things. And that’s what I like doing.” (Tom - living with dementia)

David provided vivid descriptions of how he regularly goes to two benches to sit alone in a natural setting:

“It’s a freedom to explore whatever emotions you have at that time with there being no pressures…It’s almost as if I walk into a fantasy land. Everything else just ceases to exist” (David – living with dementia).

Tom and David outline their appreciation of connecting to nature as a deeply personal experience without the interruption of others. These findings demonstrate a degree of incongruence with Noone and Jenkins (2018) and Smith-Carrier et al. (2021) on social connectedness benefits of being in nature.

Conversely, some participants limited their time in nature due to negative bodily sensations they experienced in adverse conditions, such as the disorientating effect of the wind, as Tom notes: “wind -that is something that has affected me more and more as my brain disease develops”. Participants also noted feeling the effects of being cold and wet more than they used to and thus tried to avoid going outside in inclement weather. This is echoed in other studies (Hendriks et al., 2016), recognising the multi-sensational role of nature and climatic constraints. This tends to reflect comorbidities rather than specifically dementia, as Paul explains in relation to fishing:

“I’ve had a stroke a few years ago and I’m on blood thinners and if it’s cold I feel the cold terribly so I’m a fair-weather fisherman. You know it’s got to be warm for me to go out.”

### Sense of self and identity

A central element of embodiment theory, ‘based on the assumption that thoughts, feelings, and behaviours are grounded in bodily interaction’ (Meier et al., 2012, p. 1), is phenomenon-based approaches and the concern with the everyday. By embracing key constructs like identity and sense of self in understanding individual differences and responses towards nature, it helps uncover the subjective feelings and sensory experience of being in nature. As Peter states, “it’s either going back to what you like doing or just sitting staring at the walls all day… I’d rather get out”, reflecting the changes to their leisure lives and isolation. Several respondents noted their lifelong associations with leisure pursuits (e.g., sailing, fishing, or walking in nature) while others expressed little interest in pursuits with which they have lifelong negative associations such as riding due to falling off a horse when younger (Henry) or always finding “fishing a bit boring” (Sarah). Although some participants derived enjoyment from learning and experiencing new things, to many, it was lifelong associations that framed their current preferences.

Places could instigate feelings of reminiscence among those living with dementia. For example, Tom states that although his family life growing up was difficult, his favourite place to visit is the beach, as he remembers the sense of enjoyment of family holidays there. Similarly, Lucy mentioned how she was looking forward to going to a park she had not been to for a long time as this would bring back all her childhood memories, making a powerful argument for the use of organised green dementia care models that promote people-centred programmes that are based on nature (Browne, 2021).

There is also a feeling of identity associated with activities and places that goes beyond a purely cognitive process. For example, David explained how even though he is no longer able to go fishing and camping “there’s still something deep in my inner soul that says you must be by water, you must be away from the crowds” suggesting an instinctive selection of leisure spaces. Similarly, Charlotte, a supporter, describes the difficulty in understanding her father’s feelings, but how in a familiar natural setting, he opened up more - his “bubble extended” illustrating the social connectedness that nature can broker. Amelia also notes how a familiar place from childhood, a local quarry, results in her husband being able to walk longer and how “going back to this place, any time, just completely transforms him” with these deep-seated lay geographies that remain in the consciousness. This indicates how an implicit knowledge residing within the body emerges through a connection to specific places in nature which alters bodily capacity and performance. These responses indicate the complex ways in which identity is situated and emerges through the corporeal, sensate, and cognitive body. This further emerges in how moments in nature invoked corporeal responses when these reactions could not be conveyed verbally. For example, Wyatt, a supporter, describes seeing the smile on his wife’s face and observing the “overall feeling of enjoying herself” when hearing bird song or visiting a bird of prey centre. As Wyatt explained, “eventually you know within a few hours she’d probably forgotten that we were there, but I didn’t really care about that, because for me I learned that everything with dementia is in the moment”. Wyatt then alludes to the value of ephemeral bodily sensations and feelings beyond cognitive memory in natural environments.

Participants also describe how the bodily connection to place emerges in their everyday leisure encounters with nature and the process of undertaking leisure outside the home. Several participants described their enjoyment of going walking and how they have ‘usual routes’ or ‘comfort zones’ if they go out by themselves. As Peter states, “most of the places I go, I could go in my sleep actually, I basically know every bump and lump in the road”. To some, an embodied knowledge of place emerges, which relies on moving the body to access deeper knowledge within oneself. This can be seen in the quotations below showing how participants responded to being temporarily disorientated in accounts of wayfinding:

“I just walked on around the field again and suddenly thought yes I know where I am, so that was alright.” (Mia – living with dementia)

“I suddenly lost the plot and couldn’t recognise how far I had to go to my house, but I tend to live by faith a bit, so I just walk on until things become a bit clearer again.” (Caroline – living with dementia)

For Mia and Caroline, an innate bodily knowledge of place helps to ensure they can reorientate themselves as they move through the environment.

### Connectivity to others

Participants’ accounts of how social connections frame their experience of nature-based activities, thereby enhancing their sociability and feelings of belonging, are well documented (e.g., Gatrell, 2013; Wensley & Slade, 2012). Sharing a nature-based experience also improved communication:

“I enjoy a walk together [with wife] rather than on my own. It’s nice not only to see nature, it’s good to talk about it as well. I mean we’ll go out and I’ll say that’s a song thrush or blackbird or whatever…it brings it more into my mind, into focus I think” (Paul)

The shared experience of nature was also articulated in the supporter interviews. Nora described how when walking with her mother they would stop and listen to the wind blowing in the trees and her mother would also point out seasonal changes in the vegetation. Similarly, Noah described, even though verbal communication is difficult, how his wife would excitedly point out a heron. Here corporeal movement with others enhances sensory stimuli and allows the person to contribute to the experience of others. Enhanced connection to others was a common theme and expressed poignantly by Scarlett (a supporter) who found being in nature facilitated a deeper connection with her husband as “it gives us time to talk when you’re away from home”. Kelvin appreciated the more casual conservations afforded through his routine walk, stating “I always meet someone who I know so it’s quite a good walk”. As such, moving through greenspace is an embodied social experience often appreciated by people living with dementia and the person supporting them.

People living with dementia frequently indicated an aversion to going somewhere new unless accompanied to provide reassurance, reducing their fear of getting confused or lost, and for physical support. Some participants relied on holding on to someone when walking on uneven terrain, creating a physical and emotional connection to the person supporting them. Conversely, this reliance on others prevents some people from engaging fully in their favourite activities. Paul explained he could not go fishing and metal detecting frequently, as “to expect all the time the family to do things is unfair. I suppose it’s not always a conscious decision. It’s almost unconscious. I don’t want to burden them”.

Other participants note how they refrain from activities over concern about how their bodily performance may be perceived. James notes how he only plays golf with people he knows, and he would feel uncomfortable playing with strangers, being unsure of their reactions. Similarly, Lucy avoids walking groups because she feels she walks too slowly. This self-awareness also reflects the stigma still attached to dementia (Herrmann et al., 2018).

Supporters commented that watching the person living with dementia closely was essential to ensuring a safe and enjoyable experience:

“I mean there’s lots of parts of the coast path that we couldn’t walk her on because her eyesight isn’t good enough and it would stress her because she’d be looking so hard at uneven surfaces that she wouldn’t enjoy the experience” (Emma)

Amelia described how she often avoids walking groups:

“A few years ago, we tried to do the Alzheimer’s Charity Walk; basically, he wasn’t able to sustain the distance, so we had to be very, very careful. I can tell when he’s getting tired or he really is struggling, so I don’t want to put him in a situation like that. I know his limitations so I would know how far to take him and where to take him for how long” (Amelia – supporter)

Supporters relied on a detailed knowledge of the bodily performance of people with dementia when undertaking leisure activities independently. Samantha described how she is comfortable running by herself, as her partner knows how fast she runs different routes, so will know where she is at any given time. Interestingly, issues of agency arise among some people living with dementia, with their ability to take risks compromised, as demonstrated by Peter and Kelvin:

“Because of my balance and my vision, people have told me look don’t be going to the towpaths. What if your balance goes and you fall in And I keep saying but what if I was crossing the road and get knocked down by a car. We’ve all gotta have risks” (Peter – living with dementia)

“They were all very protective you know like they were following me round the campsite! You know, stuff like that and I’m like, ‘what you doing?’, but then they got the message and just left me to it”. (Kelvin – living with dementia)

The accounts of Peter and Kelvin demonstrate the notion of leisure resistance and restrictions of others and the controversy associated with surveillance (Kontos & Martin, 2013).

## Discussion

This paper is among the first to explore embodied experience of nature-based and outdoor activities among people with dementia living in the community and informal carers of people with dementia. This addresses a research gap, as most previous studies in this area have focused on people with dementia in residential or day care settings and the leisure lives of people living at home with dementia are still little explored by researchers, although there is an emergent interest in this focus as Gray et al. (2024) indicate. Our study develops several distinct contributions to knowledge. First, it confirms some of the findings from the grey literature (e.g., Mapes et al., 2016) that people living with dementia can have rewarding, pleasurable and stimulating leisure lives, enhanced by engagement with nature. Second, we demonstrate that leisure lives do adapt, as they do in the general population throughout the life course, where challenging activities and pursuits may be replaced with more achievable outcomes. This is testimony to a positive, enabling approach where barriers and constraints to leisure are negotiated and overcome to achieve continuity in the leisure experiences of people with dementia post-diagnosis. Third, the study shows that a dementia diagnosis does not render leisure lives redundant: to the contrary, it may deepen people’s experience as they savour ‘moments’ (see Keady et al., 2020) in nature, finding greater meaning and comfort from sharing nature experiences to create social connectedness and family bonding. Fourth, the findings show that this approach to thinking may help challenge the inevitability of time-space compression, allowing outdoor experiences to be substituted nearer to home to retain familiarity and resonance with lived experiences of nature that can continue throughout a person’s life. Fifth, this study builds on the extant literature, arguing that organised programmes for people affected by dementia are the tip of the iceberg, as leisure spans their home and outdoor environments. Applying a therapeutic landscape lens illustrates the value of nature to QoL (Doughty, 2018). Bell et al. (2018) argued that such landscapes may have a healing value as well as a social, spiritual and symbolic meaning to people, and the relevance of this for people with dementia emerges from the embodied research on nature reported in this paper.

This study suggests the importance of a more holistic understanding of how people living with dementia construct leisure spaces in everyday encounters with nature. Engagement with nature can be a liberating experience, has great potential for inclusivity if accessible (type and extent of experience permitting), to most abilities. Our findings reaffirm Richardson et al. (2021, p. 23), that engaging with nature through simple activities often provides the greatest pleasure and happiness where “nature connectedness itself—tuning into nature—is a core psychological need and basic component of a good life”. Embodiment moves beyond simplistic associations between access to nature and QoL to consider how nature encounters unfold as personalised experiences that are negotiated through a process of doing, a common metaphor used in the interviews. This is important as dementia research tends to provide overly positive accounts suggesting access to nature is naturally conducive to QoL. However, as Bell et al. (2014) note, people access or derive feelings of well-being from nature in different ways, and this fluctuates in relation to shifting life circumstances and personal identities (and climatic constraints). The argument of Mapes et al. (2016, p. 10) that people living with dementia are only interested in the present and future rather than what they did in the past is challenged by this paper, as past experiences, social relations, and bodily capacities uniquely frame how people living with dementia experience nature-based pursuits. These experiences span a continuum ranging from little interest in nature to finding nature crucial for living well with dementia. Evidence exists of people withdrawing from groups and pursuits, not because of their capacities, but because of how their capacity is assessed by others, reflecting a ‘darker side’ to therapeutic spaces (Bell et al., 2018) which can exclude certain bodies or deem others out of place. This resonates with the leisure literature on how ethnic minority and disabled groups are under-represented more generally in outdoor leisure (see Burgess, 1996; Burns et al., 2009; Comber et al., 2008; Neal, 2002; Rishbeth, 2001; Tregaskis, 2004). Dementia may be added to this list in settings where people feel the need to withdraw from participating due to the effects of dementia or the attitudes of others.

### Implications

Embodiment, as Downs (2013) indicated, opens new avenues for research. It helps us deconstruct how people living with dementia frame their experience of nature-based activities. This knowledge can contribute to the design of dementia-inclusive experiences targeting diverse needs. For visitor economy businesses and organisations, the diversity in personal characteristics and lived experience of dementia means one size does not fit all. As Richardson et al. (2021) illustrate, it may be the simplest of innovations or adaptations that makes a major difference, such as providing a bench to sit on and mindfully absorb the *moment*. Recognising the value of facilitating immersive experiences entails recognising the deeply personal ways in which people living with dementia connect, construct meaning, and derive value from nature through their bodies. It provides new opportunities for dementia practitioners and businesses to think creatively about the types of nature-based pursuits people may be interested in, why and what they want from them. To practitioners this may appear as passive and low key but that may mean a great deal to the participant. We need to reframe leisure through the eyes of the participants and not presuppose what people want and need, just as highly successful customer-centric organisations do. People living with dementia have leisure lives and nature helps them to seek meaning and well-being benefits when they choose to engage with it in the way they want to. That engagement recognises an increased awareness and understanding of individuals continued capabilities and capacities that can facilitate participation.

### Limitations

Our study relied on the reflections of people living with dementia and their supporters about their pursuits in nature. To ensure we provided a faithful account of this aspect of living with dementia, and to triangulate our data with a reference group, we presented an overview of our findings to our project advisory group consisting of people living with dementia and carers, who supported the conclusions drawn. We did not set out to encompass the heterogeneity of those living with dementia in the community and we did not restrict participation to any specific stage of cognitive impairment. During the recruitment of participants, we were mindful of diversity but, despite our best efforts, our sample consists of people who mostly identify as White British. While Low et al. (2019) and Brijnath et al. (2022) pointed to the need to expand the diversity of participants in dementia research, we recognise our findings may not apply to individuals who identify as belonging to a different social or ethnic group. Yet our research does begin to illustrate how the findings on organised programmes and interventions using nature are not applicable to the diversity of leisure lives which people living with dementia have. Our data has allowed for exploration of key themes across age groups, geographic locations, and types of participants. We recognise there may be significant variation in experiences of nature-based pursuits depending on one’s degree of cognitive impairment which we did not seek to measure or control for.

### Conclusions

This study provides an in-depth account of the experiences of people living with dementia participating in everyday nature-based pursuits in their leisure time. The contribution to knowledge emerges in the intersection between nature, people living with dementia, their carers and supporters and the providers or gatekeepers controlling access to nature (where applicable). It is evident from the findings that people living with dementia have differing levels of agency that they express in their leisure lives, a feature often overlooked. Organised events may only fill a fraction of the weekly time budgets people with dementia have for leisure and so more regular engagement with the outdoors needs to be fostered. Nature, and the connections it offers, are very wide ranging: from reminiscing and recollecting past experiences of the place, through to living well in the present, and hope for a fulfilling future. As cognitive challenges are frequently experienced alongside other comorbidities affecting physical health, it is important to understand how people living with dementia experience nature-based pursuits. Nature-based pursuits can instigate bodily sensations and feelings which are valued beyond a purely cognitive process and may have deep-seated emotional meaning irrespective of any cognitive or communicative difficulties a person may be experiencing. Nature is experienced and valued in deeply personal ways, and this research indicates that expressing individual preference and exploring capabilities through nature-based experiences and activities promotes agency. For practice, this requires a reframing of nature-based activity for people living with dementia away from an association with care or therapy to one of individual autonomy. Recognising, supporting, and facilitating people living with dementia to lead fulfilling leisure lives is likely to become a growing focus for researchers, thus emphasising the importance of understanding the leisure domain in a more holistic, interconnected, and seamless way. Addressing this invisibility of the leisure lives of people with dementia through more sustained programmes of research will enable us to promote the well-being benefits of out-of-home leisure and help people to live well with dementia.

## Note

1. Unfortunately the ONS (2017) Leisure time in the UK report does not permit a disaggregation of engagement with nature as it is distributed across multiple categories of activity.
